# The health and wellbeing of Australian farmers: a longitudinal cohort study

**DOI:** 10.1186/s12889-016-3664-y

**Published:** 2016-09-15

**Authors:** Bronwyn Brew, Kerry Inder, Joanne Allen, Matthew Thomas, Brian Kelly

**Affiliations:** 1 Bathurst Rural Clinical School, Western Sydney University, Bathurst Base Hospital, Howick St, Bathurst, NSW Australia; 2 Centre for Rural and Remote Mental Health, University of Newcastle, Forest Road, Orange, NSW Australia; 3 Medical Epidemiology and Biostatistics Department, Karolinska Institute, Stockholm, SE-171 77 Sweden; 4 School of Nursing and Midwifery, University of Newcastle, University Drive, Callaghan, NSW Australia; 5 Massey University, Private Bag 11222, Palmerston North, New Zealand; 6 Centre for Brain and Mental Health Research, and School of Medicine and Public Health, University of Newcastle, University Drive, Callaghan, NSW Australia; 7 School of Psychology, Charles Sturt University, Panorama Drive, Bathurst, NSW Australia

**Keywords:** Farmers, Mental health, Wellbeing, Rural, Epidemiology

## Abstract

**Background:**

Isolation, long work days, climate change and globalization are just some of the many pressures that make farming a vulnerable occupation for incurring mental health issues. The objective of this study was to determine whether farming in Australia is associated with poorer wellbeing, physical and mental health, and less health service use.

**Methods:**

The Australian Rural Mental Health Study, a longitudinal cohort study was analysed over four time points comparing farmers with non-farming workers (*n* = 1184 at baseline). Participants were recruited from rural NSW, Australia. A number of physical, mental health, wellbeing, service use outcomes were assessed using generalised estimating equations including all waves in each model. Barriers to seeking help were also assessed.

**Results:**

Farmers who lived remotely reported worse mental health (β −0.33, 95 % CI −0.53, −0.13) and wellbeing (β −0.21(95 % CI −0.35, −0.06) than remote non-farm workers regardless of financial hardship, rural specific factors eg drought worry, or recent adverse events. All farmers were no different to non-farming workers on physical health aspects except for chronic illnesses, where they reported fewer illnesses (OR 0.66, 95 % CI 0.44, 0.98). All farmers were half as likely to visit a general practitioner (GP) or a mental health professional in the last 12 months as compared to non-farm workers regardless of location (OR 0.59, 95 % CI 0.35, 0.97). Rural workers felt that they preferred to manage themselves rather than access help for physical health needs (50 %) or mental health needs (75 %) and there was little difference between farmers and non-farm workers in reasons for not seeking help.

**Conclusions:**

Remoteness is a significant factor in the mental health and wellbeing of farmers, more so than financial stress, rural factors and recent adverse events. Creative programs and policies that improve access for farmers to GPs and mental health professionals should be supported.

**Electronic supplementary material:**

The online version of this article (doi:10.1186/s12889-016-3664-y) contains supplementary material, which is available to authorized users.

## Background

‘Farmers are almost unique as a group whose work is so intimately tied with every aspect of their lives and the lives of their families, often across several generations’ (Gregoire [1], p 472).

Farmers work long hours, have physically demanding work, are often isolated socially and geographically from services, are less likely to take vacations and less likely to retire than people in other occupations [2, 3]. In addition, farming has suffered recent pressures in the form of; globalization, economic rationalisation [4], pest and disease outbreaks [5], diminishing rural populations [3, 4], drought [6, 7] and climate change [8]. Therefore farmers could be considered to be a vulnerable population and the association between work and health is particularly pertinent for their livelihood and wellbeing.

In Australia, two-thirds of the land is used for farming, 90 % of this is for livestock grazing particularly cattle and sheep. Australian farmers supply around 93 % of Australia’s food as well as supplying many profitable export industries. In regional and remote (New South Wales) NSW, where this study is set, the main farming industries are beef cattle, sheep and grain crops [9, 10].

### The physical health of farmers

Research into the mortality rates of farmers compared to national averages varies according to region. Scandinavian and US research have found mortality rates are lower in farmers than other residents [11–13], conversely, Australian and UK research has found that mortality rates are higher for farmers [14, 15]. These inconsistencies may reflect differences in farming practices as well as political or geographic aspects impacting on health. Countries where farmers have lower mortality also have lower risk factors such as less smoking, more exercise, and less alcohol consumption [2, 12]. Furthermore, in Australia, the physical health of the rural population in general is poorer than the urban population [16], therefore it is possible that higher morbidity and mortality rates for farmers in Australia are not unique but are simply indicative of the rural situation. Indeed some studies have found that farmers are no different in physical health and risk factors to the non-farming rural population [17, 18] whereas others have found that Australian farmers have higher alcohol consumption and do less physical exercise [19, 20].

### The mental health of farmers

Suicide rates in farmers are higher than most other occupations and national trends [21–23]. In contrast, a number of studies have found that the mental health of farmers is no different to other rural and urban residents [19, 20, 24, 25]. Several explanations have been given for this discrepancy such as farmers having greater access to means for suicide [26], farmers are unwilling or unable to acknowledge mental health problems and seek help [25, 27], or that farmers may have a lower threshold for suicide than other people in the setting of psychological stress [25]. A recent study of subsidised mental health services in Australia found that usage rates decreased with increasing remoteness [28]. This suggests that remoteness is associated with inequality in the health care system resulting in less access to services [28], but may also indicate that remote rural residents are less inclined to seek help and are reluctant to acknowledge problems [29, 30].

Overall, there is a paucity of data regarding the physical and mental health of farmers compared to their rural counterparts. It is important for health service providers to assess the health needs of those in farming and whether the issues surrounding mental health are specific to farming or are characteristic of rural areas in general.

### The Australian Rural Mental Health Study (ARMHS)

Previous research on the Australian Rural Mental Health Study (ARMHS) by Fragar et al. found that at baseline 34% of farmers and farm managers suffered moderate to high levels of distress, which was not dissimilar to other occupations such as teachers, clerical, sales, community and personal service workers but was higher than other types of managers in rural areas [24]. The current study is expanding on the previous study by Fragar et al. by looking at all four waves of data and including other mental health variables such as self-report mental health and current depression, as well as physical health and service use variables to build an overall picture about farmer health. This is a secondary data analysis based on a subgroup of workers.

The objective of this study was to use a longitudinal rural cohort study to determine whether the farming occupation is associated with poorer wellbeing, physical and mental health, and less health service use compared with other rural workers. Secondary objectives were to investigate whether differences in mental, physical health outcomes and service use could be explained by rural-specific mediating factors (eg financial hardship, drought worry, or rural-specific barriers to service use).

## Methods

The Australian Rural Mental Health Study (ARMHS) was designed to improve our knowledge of the determinants and outcomes of common mental disorders in rural and remote communities by surveying those people who live in these localities in New South Wales (NSW), Australia. Patterns and determinants of service use were identified, with reference to the diverse social and community factors [31, 32]. The ARMHS is a longitudinal cohort study that followed participants over four waves at baseline, year 1, year 3 and year 5. Complete details of the methodology and measures used in the ARMHS, a longitudinal population-based cohort, are provided elsewhere [31, 32].

### Recruitment

Sixty Local Government Areas were identified from three Australian rural health service regions in NSW, representing approximately 70 % of the geographic region of nonmetropolitan NSW. Local government areas were classified using the Australian Standard Geographic Classifications (ASGC) based on the Accessibility/Remoteness Index of Australia (ARIA) + [33]. An equal number of households was identified for recruitment across the ARIA categories, given that population density decreases with remoteness this led to over-sampling of the remote and very remote local government areas which helped to ensure adequate representation of these regions. The baseline sample were invited to participate in the study between 2007 and 2009 from households identified randomly from the Australian electoral roll. The baseline survey used self-report measures, administered by post in two parts (survey A and B) mailed 2 weeks apart. Additional questionnaires were sent to all originally recruited participants at 1, 3 and 5 year follow-up waves. These questionnaires contained similar questions to those at baseline except for exercise habits which were only included at baseline. Only those over 18 years of age were included in the study. Sample bias has been outlined previously [31, 32], in brief, younger people in general, the oldest age group within inner regional areas, and younger people in very remote regions were among the most difficult to contact.

### Definition of the study group

A previous study on psychological distress by occupation in ARMHS participants, found that those unemployed and those permanently unable to work had the highest distress rates and those who were retired had the lowest rates [24]. As the ARMHS cohort has a large retired group (*n* = 796) and retirees, unemployed and permanently unemployed are represented unequally in the farmer/farm workers compared to non-farm workers, these groups were excluded from the study. Those who identified as studying or being in a care-giving role were also excluded, leaving those in paid employment. ‘Farmers’ were those who identified as being a farmer or farm manager. Farm residents were defined as those who reported living on a farm but who were employed elsewhere (not as a farmer). Other rural workers were defined as those who did not identify ‘farmer’ or ‘farm manager’ when asked for their occupation and who were in paid employment in a rural area.

### Variables and outcomes

#### Socio-demographic characteristics

Basic socio-demographic questions were included at baseline: *age in years; gender; education*; *years living in a rural district* and *marital status. Financial hardship* was assessed using the perceived prosperity item from the Household, Income and Labour Dynamics in Australia (HILDA) study (defined as ‘very poor’, ‘poor’ or ‘just getting along’ vs ‘reasonably comfortable’, ‘very comfortable or ‘prosperous’) [34]. *Remoteness* of location of residence was measured using the continuous variable ARIA+, developed by the National Centre for Social Applications of Geographic Information Systems (http://www.gisca.adelaide.edu.au) and ASGC [33]. ARIA + is an index of remoteness derived from measures of road distances between localities and service centres, ranging from 0 (high accessibility) to 15 (high remoteness). An ASGC ranking of ‘inner or outer regional’ areas correlates to an ARIA+ index value of 0.2 to 5.92, a ranking of ‘remote or very remote’ correlates to an ARIA+ index value of 5.93 to 15. *Community socioeconomic advantage* was measured using the Australian Bureau of Statistics’ (ABS) Index of Relative Socio-Economic Advantage and Disadvantage (SEIFA) which is a standardised score of disadvantage (low values) to advantage (high values) based on collation of household level census data [35].

#### Vulnerabilities and rural mediating factors

*Recent adverse life events* were assessed using the List of Threatening Experiences [36], a 12-item count of events in the past 12 months. Trait *neuroticism* was assessed using seven items from a brief version of the Eysenck Personality Inventory, (range 0–7) [32]. The *Community and Personal Support* variable is a composite measure of the following scales: perceived availability of social support, social networks, sense of community and community participation [37]. Higher scores indicate higher levels of support (range −3.46 to 1.18). *Drought Stress* was measured using a single Likert scale item rating level of worry about drought (range 1–5, low to high). *Infrastructure and Services Perception* was assessed on common concerns in rural communities: access to health care or other services, concerns regarding fuel prices, people moving in or out of the district, scored on 5-point Likert scales (range 1–5, low to high). *Sense of Place* is a composite measure representing connection with local environment and landscape scored on five point Likert scales (range 10 to 50, low to high) [38].

#### Physical health outcomes

Overall physical health was measured using a single *self-report physical health* question (range 1 ( poor)–5 (excellent)). *Harmful alcohol use* in the last 6 months (baseline) or 12 months (1,3 and 5 year waves) was measured using the Alcohol Use Disorder Identification Test (AUDIT-10), maximum total score of 40 [39]. *Overweight* was measured by using height and weight measurements obtained through self-reported survey responses and corrected for reporting biases using equations based on 2007–2008 Australian national survey data [40]. Body Mass Index (BMI) was calculated as weight in kilograms divided by height in metres squared, values ≥ 25 used to classify overweight. *Adequate exercise* is an indicator of whether reported activity levels met Australian Government Department of Health Exercise Guidelines (http://www.health.gov.au/internet/main/publishing.nsf/content/health-pubhlth-strateg-phys-act-guidelines#apaadult). For an adult <64yrs: 2.5–5 h per week of moderate intensity physical exercise OR 1.25–2.5 h per week of vigorous physical activity. For those >65 years: 30 mins per day (on most days) of moderate intensity physical activity. *Chronic illness* represents the lifetime diagnosis of at least one chronic disease (stroke, heart attack/angina/heart disease, cancer, diabetes). *Injury in last 12 months* refers to only those injuries requiring hospital treatment in the last 12 months.

#### Mental health and wellbeing outcomes

*Psychological Distress* was measured using Kessler 10 (K10), a 10-item self-report questionnaire that assesses the frequency of psychological distress over the past 4 weeks with higher scores denoting greater psychological distress (‘low’ <16, ‘moderate’ 16–24, ‘high’ > 24) [31, 41]. *Current Depression* was measured using the 9-item patient health questionnaire (PHQ-9) assessing depressive symptoms in the past 7 days (5, 10, 15 and 20 represent mild, moderate, moderately severe and severe depression respectively ) [42]. The *Well-being Index* included seven scores in an aggregate measure: overall K10 score, days out of role in the past month, overall physical health and mental health, ability to perform everyday duties and tasks, satisfaction with relationships, and overall satisfaction with life [32]. Higher scores indicate greater well-being, with the zero point on the standardised scale reflecting the average baseline response from all ARMHS participants. Overall mental health was measured using a single *self-report mental health* question (range 1 (poor)–5 (excellent)).

#### Service use

*Number of visits to General Practitioners (GPs) in the last 12 months* was measured in categories; none, 1–2 times, 3–4 times, 5–6 times, 7–9 times, 10–12 times, 13+ times. *Sought Help for a Mental Health (MH) Problem from a professional in the last 12 months* (Yes/No) included; GPs, psychiatrists, psychologists, drug and alcohol counsellors. *Perceived barriers to seeking health care for physical or mental health* included 11 barriers each rated on a five point Likert scale (‘not at all’ to ‘a lot’) and grouped into three broad categories which were developed using principal component analysis: attitudinal barriers (eg ‘I prefer to manage myself’, ‘I was afraid to ask for help or what others may think’); structural barriers (eg ‘It is too far to travel’, ‘I couldn’t afford to pay for the service’, ‘It takes too long to get an appointment’) and time barriers (eg ‘I can’t get time away from work’, ‘I care for someone else’) [43].

### Statistical methods

Data entry, cleaning, aggregation and analysis were conducted using the Statistical Package for Social Sciences (SPSS version 17.0; Chicago, IL, USA). Initial analyses compared demographic factors, specific rural concerns, and vulnerabilities of farmers, farm residents (employed elsewhere) and other rural workers at baseline. T-tests and chi square tests were used for initial analyses. Significance was set at *p* < 0.05.

As the primary aim of this study was to examine factors influencing the health of people who live and work on farms, farm residents employed elsewhere were grouped with other rural workers as ‘*non-farm workers’* for subsequent analyses. Generalised estimating equations (GEE) were used to compare the physical health, mental health, distress, wellbeing and service use outcomes of farmers with non-farm workers over time (Baseline, Year 1, Year 3 and Year 5). GEEs were used so that all data points could be included no matter when the rural workers participated in the survey or how many waves they participated in. This was to maximise the power for the study. Directed Acyclic Graphs (DAGs) were created to assess the causal relationships between covariates (see Additional file 1) for use in the GEEs (Additional file 1) [44]. Covariates and their associations with the outcome, independent variable and other covariates were added to the DAG as informed by literature on farming, mental and physical health [1, 4, 6, 19]. The DAGs were created using online DAGITTY (version 2.2, Johannes Textor) software, http://www.dagitty.net/. Based on the DAGs, age, gender, education level and remoteness (ARIA+) were identified as potential confounders for all analyses (as recorded at baseline). The other covariates were identified as mediating factors (those factors that are on the causal pathway between the exposure, farming, and the outcome and may mediate or modify the effect of farming status on the outcome) and varied for each of the outcomes (see Additional file 1: Figures S1–S4). The mediators included rural specific factors and vulnerabilities.

Unadjusted GEEs were performed for farming status alone, then an adjusted model was run with only the confounders (TOTAL effect). Since level of isolation and remoteness is an important part of rural health outcomes, the remoteness of participant’s location (ARIA+) by farmer status was included as an interaction term in each model. If this term was significant (*p* < 0.05) then the analysis was stratified by remoteness (‘inner and outer regional’: ARIA+ values 0.20–5.92; ‘remote and very remote’: ARIA+ values 5.92–15.00). A further GEE model was run including mediators (DIRECT effect, results shown in Additional file 1: Tables S1 and S2) [44].

Barriers to accessing health care for physical and mental health were assessed for farmers compared with non-farm workers using 3 and 5 year data only as the earlier waves did not differentiate between mental and physical health. Averages were calculated for each category and GEE analysis was applied to compare the barriers for farmers and non-farm workers. Due to a skip question in the survey only a proportion of participants answered the mental health needs barrier questions (those who answered ‘no’ to ‘Do you think you got as much help as you needed?’).

## Results

**Fig. 1:**
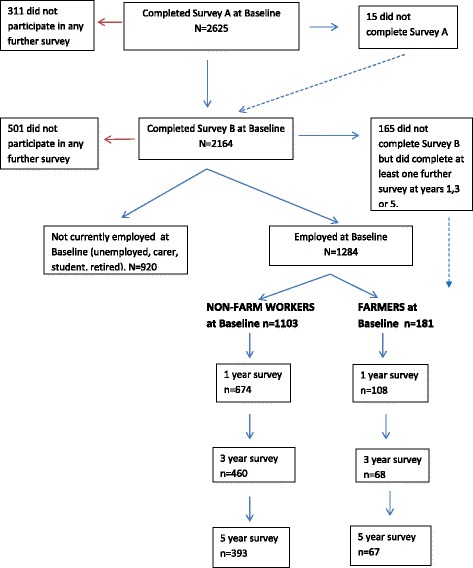
Participation in ARMHS by Employment status, Baseline to Year 5

The baseline cohort comprised *n* = 2639 individuals who consented to participate. Full details of the study sample are described by Kelly et al. [31]. At years 1, 3 and 5 the participation rates were 71 % (*n* = 1702), 48 % (*n* = 1262), 44 % (*n* = 1158) respectively. After excluding those not currently employed at baseline the number for baseline analyses was *n* = 1284 (Fig. 1), this included 181 farmers and 1103 non-farm workers (other rural workers and farm residents employed elsewhere). At the 1, 3 and 5 year waves respectively, 782 (61 %), 528 (41 %) and 460 (36 %) employed persons remained in the study (Fig. 1). For longitudinal analyses there were 3055 data points available as participants contributed multiple time points of data.

A comparison of demographic characteristics of those included in the study at each time point (Table 1) indicated that none of the demographics differed statistically between waves excepting those with financial hardship were slightly more likely to drop out of the study over time (*p* < 0.01). Those who lived remotely were not more able to drop out. A comparison of the main outcomes over each wave indicated that self report physical health declined over the study, which could be an result of aging. However, self report mental health and the wellbeing index did not vary significantly over time (Table 1).

**Table 1:** Demographic and main outcome comparison of all included participants at baseline with those remaining in the study at 1, 3 and 5 years

	Baseline	1 year	3 years	5 years
	*N* = 1284	*N* = 782	*N* = 528	*N* = 460
Demographics				
Age (mean, SD)	48.3 (11.9)	50.7 (11.2)	51.8 (10.4)	53.9 (10.5)
Gender, female (n, %)	733 (57 %)	460 (59 %)	317 (60 %)	274 (60 %)
Married/defacto (n, %)	1019 (79 %)	628 (80.7 %)	435 (82.4 %)	373 (82.5 %)
Completed highschool (n, %)	1000 (80.3 %)	619 (82.4 %)	435 (84.5 %)	376 (84.9 %)
Financial hardship (n, %)*	289 (29 %)	191 (25 %)	116 (22 %)	102 (22.5 %)
ARIA + (mean, SD)	4.47 (3.35)	4.34 (3.27)	4.44 (3.40)	4.26 (3.22)
Main Outcomes				
Self-Report Physical Health (mean, SD)*	3.45 (0.96)	3.24 (0.91)	3.24 (0.91)	3.27 (0.91)
Self-Report Mental Health (mean, SD)	3.60 (0.99)	3.54 (0.93)	3.57 (0.93)	3.48 (0.97)
Wellbeing Index (mean, SD)	0.06 (0.69)	0.03 (0.63)	0.06 (0.62)	0.01 (0.64)

*p < 0.01 (ANOVA)

### Characteristics of farmers, employed farm residents and other rural workers at baseline

Comparison of demographic factors by group found that farmers differed from other rural workers on all scales (Table 2). Farmers were more likely to be older, male, married, suffering financial hardship, have lived in a rural area for longer, live more remotely and in an area of disadvantage. Half the farmers at each wave were from remote and very remote areas (*N* = 91, 51, 34, 31 for baseline, year 1, year 3 and year 5 respectively). Those who were farm residents but employed in other occupations were more similar to other rural workers except they were more likely to be married and live in an area of disadvantage, therefore farm residents employed in other occupations and other rural workers were grouped for subsequent analyses (non-farm workers).

**Table 2:** Baseline Demographics, Vulnerabilities and Rural modifiers of all those Employed: Farmers, Farm residents (employed elsewhere) and Other rural workers

	Other rural workers	Farm Residents^a^	Farmers^b^
N	850	253	181
Age (mean, SD)	47.6 (11.5)	47.0 (12.0)	52.8 (12.6)**
Gender Female (n, %)	495 (58.2 )	167 (66.0)*	68 (37.6)**
Married/defacto (n, %)	628 (74.0)	207 (81.8)**	155 (86.6)**
Completed High school (n, %)	667 (81.2)	201 (81.3)	128 (74.4)*
Financial Hardship (n, %)	181 (27.3)	66 (29.8)	47 (36.4)*
Years Rural (mean, SD)	35.66 (16.2)	34.27 (16.52)	44.21 (15.94)**
ARIA+ (mean, SD)	4.25 (3.35)	3.96 (2.76)	6.12 (3.61)**
SEIFA (mean, SD)	945.6 (34.5)	934.5 (36.83)**	921.69 (30.45)**
Vulnerabilities			
Number of Adverse Life Events (mean, SD)	1.52 (1.53)	1.49 (1.48)	1.67 (1.64)
Neuroticism (mean, SD)	2.08 (1.92)	1.93 (1.74)	1.91 (1.78)
Community and Personal Support (mean, SD)	−0.06 (0.67)	0.10 (0.63)**	0.06 (0.65)*
Rural Modifying factors			
Drought stress (mean, SD)	1.20 (0.40)	1.40 (0.49)**	1.65 (0.48)**
Infrastructure and Services Perception (mean, SD)	2.40 (0.95)	2.43 (0.93)	2.71 (0.95)**
Sense of Place (mean, SD)	34.35 (6.19)	36.89 (6.15)**	38.27 (6.91)**

^a^Farm residents (employed elsewhere) compared with Other rural workers

^b^Farmers compared to Other rural workers

*p ≤ 0.05, **p ≤ 0.01

Comparison of vulnerabilities at baseline found that farmers and other rural workers did not differ on number of life events and neuroticism (Table 2). Farmers were found to score higher on rural specific variables such as drought stress, concerns about infrastructure and services, and a higher sense of place, than other rural workers.

### Longitudinal analysis. Physical and mental health outcomes of farmers compared to non-farm workers

Univariate GEE analyses over 5 years found that farmers were more likely to have poorer self-reported physical health estimate than non-farm workers (Table 3), reporting an average 0.14 (95 % CI −0.27, −0.02) points lower. The adjusted analyses found that regional farmers did not differ in self-reported physical health to regional non-farm workers but that those living in more remote regions were more likely to rate their physical health poorer by 0.19 points (95 % CI −0.39, 0.01) , although the significance level was borderline 0.06. The adjusted analysis (Table 3) did not find any differences between farmers and non-farm workers for alcohol consumption, smoking, being overweight or recent injury requiring treatment. However, farmers were less likely to have a doctor diagnosed chronic illness in the last 2 years (adjOR 0.66, 95 % 0.44, 0.98) than non-farm workers. Adding mediators to the GEE such as financial hardship and injury in the last 12 months did not greatly change the magnitude of the differences between farmers and non-farm workers on all physical outcomes (Additional file 1: Table S1). However, the association between farmers and self-reported physical health and chronic health became non-significant (physical health −0.17, 95 % CI −0.38, 0.04 (for remote), chronic illness 0.66, 95 % CI 0.43, 1.02). This could be a loss of power due to missing data on some mediating variables, rather than showing that the mediators themselves influenced the association.

**Table 3:** General Estimating Equation (GEE) Longitudinal analysis over 5 years for Physical Health outcomes and General Practitioner (GP) Service use. Farmers v’s Non-farm Workers. Results show Beta values for continuous variables and odds ratios (OR) for dichotomous variables (95 % Confidence Intervals)

	Farmers (estimated marginal means)	Non-farm workers (estimated marginal means)	Unadjusted GEE Farmer v Non-farm Worker	TOTAL effect^a^ GEE Farmer v Non-Farm Worker		p Interaction^b^
				ASGC regional (inner or outer) *N* = 2112	ASGC remote (remote or very remote) *N* = 824	
Self-report Physical Health *N* = 3035	3.21 (SE 0.06)	3.35 (SE 0.02)	*β* = −0.14 (−0.27, −0.02)*	*β* = 0.03 (−0.13, 0.19)	*β* = −0.19 (−0.39, 0.01) *p* = 0.06	0.02
Harmful Alcohol Use *N* = 2750	4.16 (SE 0.28)	4.40 (SE 0.13)	*β* = −0.24 (−0.85, 0.38)	*β* = −0.44 (−1.11, 0.24)		NS
Current Smoker *N* = 2732	9 % (SE 2 %)	13 % (SE 1 %)	OR 0.64 (0.37, 3.09)	OR 0.63 (0.35, 3.71)		NS
Overweight BMI ≥ 25 *N* = 1920	71 % (SE 4 %)	68 % (SE 1.5 %)	OR 1.19 (0.81, 1.72)	OR 0.77 (0.51, 1.17)		NS
Adequate Exercise *N* = 965^c^	15 % (SE 3 %)	19 % (SE 1.4 %)	OR 0.72 (0.42, 1.22)	0.78 (0.44, 1.38)		NS
Chronic Illness *N* = 2990	14 % (SE 2 %)	12 % (SE 0.8 %)	OR 1.15 (0.80, 1.66)	OR 0.66 (0.44, 0.98)*		NS
Injury in the last 12 months *N* = 2992	18 % (SE 2 %)	17 % (0.8 %)	OR 1.07 (0.78, 1.46)	OR 1.05 (0.76, 1.45)		NS
Number of visits to a GP in the last 12 months *N* = 2693	1.46 (SE 0.08)	1.60 (0.04)	*β* = −0.09 (−0.21, 0.03)	*β* = −0.13 (−0.25, −0.01)*		NS

^a^TOTAL effect adjusted for confounders: age, gender, completed school, ARIA

^b^Probability of interaction farmer status x ARIA+

^C^This data was only recorded at baseline and therefore is not longitudinal

*p < 0.05

All farmers were less likely to have visited a GP in the last 12 months (adj β = −0.13 of a category, 95 % CI −0.025, −0.01) than non-farm workers. Adding potential mediators to the model strengthened the association (adj B = −0.16 of a category, 95 % CI −0.27, −0.04) suggesting that being a farmer has a direct effect on choosing not to go to the doctor and that this was not mediated by financial hardship, injury or physical health issues (Additional file 1: Table S1).

For self-reported mental health, farmers reported an average of 0.16 adj (95 % CI −0.29, −0.02) points lower than non-farm workers (Table 4). After stratifying for remoteness, regional farmers showed no difference for self-reported mental health compared to regional non-farm workers, however remote farmers reported worse self-reported mental health by 0.33 points adj (95 % CI −0.53, −0.13) compared to remote non-farm workers. Similarly, remote farmers had a lower wellbeing index by 0.21 points adj (95 % CI −0.35, −0.13) compared to remote non-farm workers (Table 4) but there was no difference for regional employees. Adding potential mediating factors in both models (the direct effect) slightly moderated the size of the effects but still found that being a farmer is directly associated with lower self-reported mental health (−0.19, 95 % CI −0.37, −0.01) and wellbeing (−0.22, 95 % CI −0.38, −0.06) in remote areas regardless of financial hardship, rural specific factors or recent adverse events (Additional file 1: Table S2).

**Table 4:** GEE Longitudinal analysis over 5 years for Wellbeing, Mental Health outcomes and Visiting a Mental Health Professional. Farmers v’s Non-farm Workers. Results show Beta values for continuous variables and odds ratios (OR) for dichotomous variables (95% Confidence Intervals)

	Farmers (estimated marginal means)	Non-farm workers (estimated marginal means)	Unadjusted GEE Farmer v Non-farm Worker	TOTAL effect^a^ GEE Farmer v Non-Farm Worker		p Interaction^b^
				ASGC regional (inner or outer) *N* = 2112	ASGC remote (remote or very remote) *N* = 824	
Wellbeing Index *N* = 3036	−0.07 (SE 0.05)	0.04 (SE 0.02)	*β* = −0.12 (−0.21, −0.02)*	*β* = −0.01 (−0.14, 0.13)	*β* = −0.21 (−0.35, −0.06)**	0.01
Self-report Mental Health *N* = 3034	3.42 (SE 0.06)	3.57 (SE 0.03)	*β* = −0.16 (−0.29, −0.02)*	*β* = 0.03 (−0.17, 0.23)	*β* = −0.33 (−0.53, −0.13)**	<0.01
Psychological distress *N* = 3032	14.6 (SE 0.33)	14.4 (SE 0.14)	*β* = 0.01 (−0.04, 0.06)	*β* = 0.03 (−0.02, 0.09)		NS
Current depression *N* = 2743	2.65 (SE 0.22)	2.91 (SE 0.10)	*β* = −0.25 (−0.74, 0.24)	*β* = −0.11 (−0.65, 0.43)		NS
Sought help from a Mental Health professional in last 12 months *N* = 2760	9 % (SE 2 %)	16% (SE 1 %)	OR 0.52 (0.32, 0.83)**	OR 0.59 (0.35, 0.97)*		NS

^a^TOTAL effect adjusted for confounders: age, gender, completed school, ARIA

^b^Probability of interaction farmer status x ARIA+

*p < 0.05, **p < 0.01

Over 5 years, farmers and non-farmers did not differ in distress levels as measured with K10 or for current depression (Table 4).

Nine percent of farmers had visited a health professional for mental health reasons compared to 16 % of non-farmers (estimated marginal means). The odds of farmers visiting a professional for mental health reasons was 0.59 adj (95 % CI 0.35, 0.97) compared to a non-farm worker (Table 4). There was no difference by remoteness. Adding mediators to the model to determine the direct effect strengthened the association to OR 0.54 (95% CI 0.31, 0.93) (Additional file 1: Table S2).

### Longitudinal analysis. Barriers to accessing health care for physical and mental health needs

**Fig. 2:**
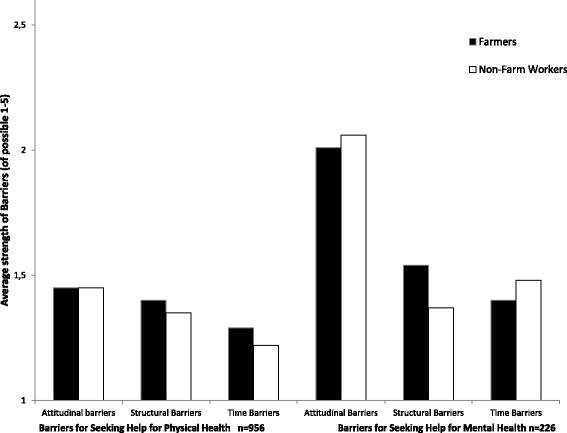
Barriers for Seeking help for Physical or Mental Health Needs, a comparison of Farmers and Non-Farm workers

Attitudinal barriers were the greatest barrier type for all rural workers and of these, ‘I prefer to manage myself’ was by far the most common barrier, and this was similar for farmers and non-farm workers (Fig. 2). Overall, 50 % of rural workers felt that they preferred to manage themselves rather than access help for physical health needs, and 75 % felt they preferred to manage themselves rather than access help for mental health needs. In addition, 41 % of all rural workers ‘didn’t think anything could help’ regarding their mental health and 30% were concerned about what others thought or that their mental health issues would not stay private. Overall, barriers to seeking help for physical or mental health did not differ between farmers and non-farm workers (results not shown). The only point of difference in barriers for farmers and non-farmers were structural barriers to seeking help for mental health. When adjusting for age, sex, remoteness and education level there was a trend towards farmers having greater structural barriers (*p* = 0.07). In particular, more farmers reported that distance and transport costs were too great (26 % of farmers v 13 % of non-farm workers).

## Discussion

The main findings of this study are that farmers in remote areas report worse mental, physical health and wellbeing than their non-farming rural counterparts, whereas regional farmers are not different to regional non-farm workers. Farmers are less likely to access health services, either a primary care general practitioner (GP) or a mental health professional than non-farm workers regardless of remoteness. Mediation analysis showed that although drought stress, community support, financial hardship, infrastructure perception and sense of place differ are more important to farmers, these aspects did not impact significantly on the associations between farmers, mental health outcomes and service use.

### Physical health outcomes

This study found that after adjusting for age, gender, remoteness and education level, farmers were less likely to be diagnosed with a chronic illness than non-farm workers. We hypothesise that lower diagnoses of chronic disease in farmers is a function of under-diagnosis due to lower GP attendance by farmers rather than a function of a better lifestyle since farmers did not differ on harmful alcohol use, smoking, overweight, injury or adequate exercise compared to non-farming rural residents. This may also explain why remote farmers were more likely to report worse physical health (borderline finding). In Australia, remote farmers are more likely to be isolated, have less access to medical care and therefore less likely to have chronic illnesses well managed. Under-diagnosis may therefore explain why the mortality and morbidity of farmers is higher than other Australians [14].

### Mental health outcomes and wellbeing

Farmers, in general, were no different to non-farm workers using validated tools for measuring mental health status (ie psychological distress and current depression). This is consistent with other Australian and international research which mainly use the K10 and similar tests [19, 25, 27]. However, this study did find that those farmers who lived more remotely had poorer self-reported mental health than non-farm workers living remotely and that this was not mediated by rural specific factors or vulnerabilities. Similar results were found for the well-being index. Therefore it appears that farmers are less likely to acknowledge their mental health issues when asked about specific symptoms but that they do recognise a poorer sense of wellbeing and general mental health, particularly for those living in remote areas. A study of British farmers by Thomas et al. found that farmers were less likely to report a psychiatric illness, but were more likely to think life was not worth living compared to other rural householders. The authors concluded that farmers are reluctant to admit problems or report severe symptoms of depression [25]. A qualitative Australian study by Judd et al. found that farmers experienced a range of stressors but had limited capacity to acknowledge or express these and were then less likely to seek help [27]. This is in concordance with our study which found that farmers were less likely to seek help for mental health problems regardless of rurality despite the fact that they reported lower mental health and wellbeing.

The analysis of barriers for seeking help for mental and physical health needs found that farmers had slightly more transport barriers than non-farm workers, but overall did not help to elucidate why farmers seek help less than their rural counterparts. Overwhelmingly the strongest barriers were attitudinal for both farmers and non-farm workers, and in particular there was a strong preference to manage health needs themselves. This may reflect a culture of stoicism or a lack of confidence in health professionals [45]. Studies on UK and Australian farmers found that they prefer to choose family, friends or other farmers for personal support and would prefer written advice or a farmers’ self-help group as a potential source of help rather than visits from a health or social worker [5, 27, 46]. Stoicism and a tendency to self-reliance could be considered to be both a protective mechanism to mental health problems or a barrier to finding help when it is needed [29].

### Strengths and limitations

The strengths of this study are its longitudinal design using data over four waves in a repeated measures analysis, which means that all data can be included even if one or more waves are missing for each person, increasing the reliability for a participant. Secondly, it uses both objective and subjective measures for physical and mental health which can be compared for different meaning. In addition, this study compares farmers with their rural counterparts rather than national or urban referents. This is an important distinction for policy and health promotion development to clarify which groups need to be targeted. Finally, using directed acyclic graphs provides an impartial methodological strength to determine which covariates are potential confounders and which are mediators in order to calculate the direct effect of farming on health outcomes and service use.

The limitations of this study are that there was considerable loss to follow up over time. This has the potential to cause selection bias particularly if those who leave the study do so for mental health reasons. We found that the mental health and wellbeing did not significantly change for those remaining in the study over time, in fact these indicators showed a decline (not-significant) which is the opposite of what would be expected if those with mental health issues refrained from partaking. The main differences in those leaving the study was that they were more likely to undergo financial hardship and more likely to have better physical health. Secondly, the barriers analysis was underpowered due to the barriers questions only being asked in waves 3 and 5, and also to a skip question in the survey capturing only those who expressed dissatisfaction in getting help for mental health. This means that; a) the number of farmers in the barriers analysis was small (*n* = 29) and b) there may be others who had reasons not to seek help from mental health professionals but did not answer yes to the skip question.

## Conclusions and recommendations

In conclusion the findings suggest significant variability among those working in farming in physical and mental health needs, reflecting locality and possibly those factors related to remoteness (eg poorer access to health care). Lower inclination to seek health care for physical and mental health problems is noteworthy. Given the international findings regarding the link between farming occupation and suicide, and the increasing rates of suicide with remoteness these findings provide further support for programs that aim to improve access to mental health related care to farmers, along with addressing attitudinal barriers to seeking help for such programs [47, 48]. Suicide prevention programs are most effective that enhance access to adequate health care among high risk individuals such as those who live remotely [49].

## Additional file

Additional file 1: Figure S1. Directed Acyclic Graph for Farming and Mental Health Outcomes. **Figure S2.** Directed Acyclic Graph for Farming and Physical Health Outcomes. **Figure S3.** Directed Acyclic Graph for Farming and Wellbeing. **Figure S4.** Directed Acyclic Graphs for Farming and Visits to the Doctor. **Figure S5.** Directed Acyclic Graph for Farming and Seeking Help from a Mental Health Professional. **Table S1.** DIRECT effect. Longitudinal analysis over 5 years for Physical health outcomes and GP service use. Farmers v’s Non-Farm workers. Results show Beta values for continous variables and odds ratios for dichotomous variables (95 % Confidence Intervals). **Table S2.** DIRECT effect. Longitudinal analysis over 5 years for Wellbeing, Mental health outcomes and Visiting a Mental Health Professional. Farmers v’s Non-Farm workers. Results show Beta values for continuous variables and odds ratios for dichotomous variables (95 % Confidence Intervals). (PDF 667 kb)

## Abbreviations

ABS: Australian Bureau of Statistics
ARIA: Accessibility/Remoteness Index of Australia
ARMHS: Australian Rural Mental Health Study
AUDIT: Alcohol Use Disorder Identification Test
BMI: Body Mass Index
DAG: Directed acyclic graph
GEE: Generalised estimating equation
GP: General practitioner
K10: Kessler 10 test
NSW: New South Wales
OR: Odds ratio
SD: Standard Deviation
SEIFA: Index of Relative Socio-economic Advantage and Disadvantage
