# Supplementary material for: The health and wellbeing of Australian farmers: a longitudinal cohort study
Source: BMC Public Health. 2016 Sep 15;16:988. doi: 10.1186/s12889-016-3664-y (PMC5025556; doi:10.1186/s12889-016-3664-y)
Supplement: Additional file 1: Figure S1. — Directed Acyclic Graph for Farming and Mental Health Outcomes. Figure S2. Directed Acyclic Graph for Farming and Physical Health Outcomes. Figure S3. Directed Acyclic Graph for Farming and Wellbeing. Figure S4. Directed Acyclic Graphs for Farming and Visits to the Doctor. Figure S5. Directed Acyclic Graph for Farming and Seeking Help from a Mental Health Professional. Table S1. DIRECT effect. Longitudinal analysis over 5 years for Physical health outcomes and GP service use. Farmers v’s Non-Farm workers. Results show Beta values for continous variables and odds ratios for dichotomous variables (95 % Confidence Intervals). Table S2. DIRECT effect. Longitudinal analysis over 5 years for Wellbeing, Mental health outcomes and Visiting a Mental Health Professional. Farmers v’s Non-Farm workers. Results show Beta values for continuous variables and odds ratios for dichotomous variables (95 % Confidence Intervals). (PDF 667 kb) [file 12889_2016_3664_MOESM1_ESM.pdf]

ADDITONAL FILE 1

Figure 1. Directed Acyclic Graph for Farming and Mental Health Outcomes

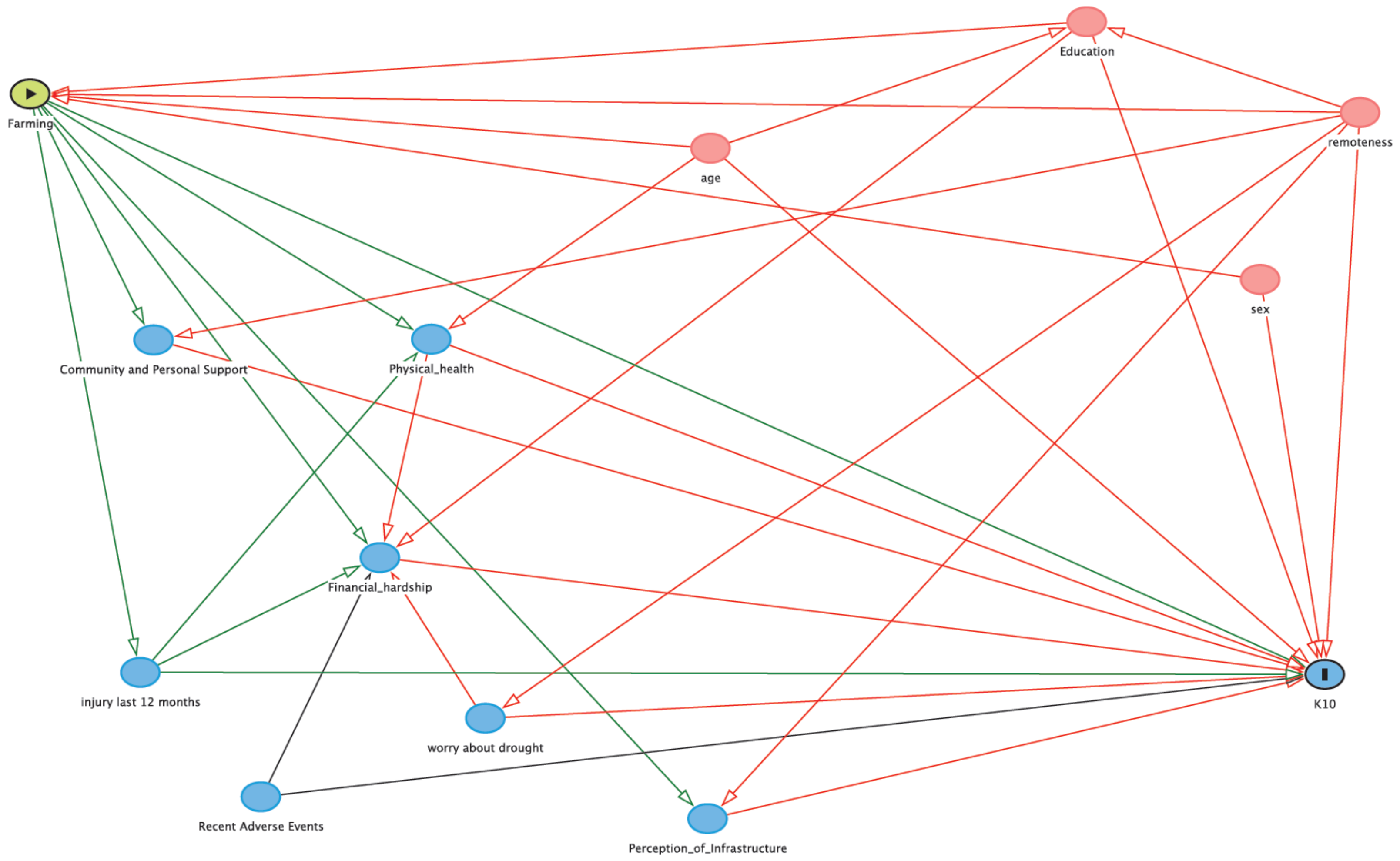

Figure 2. Directed Acyclic Graph for Farming and Physical Health Outcomes

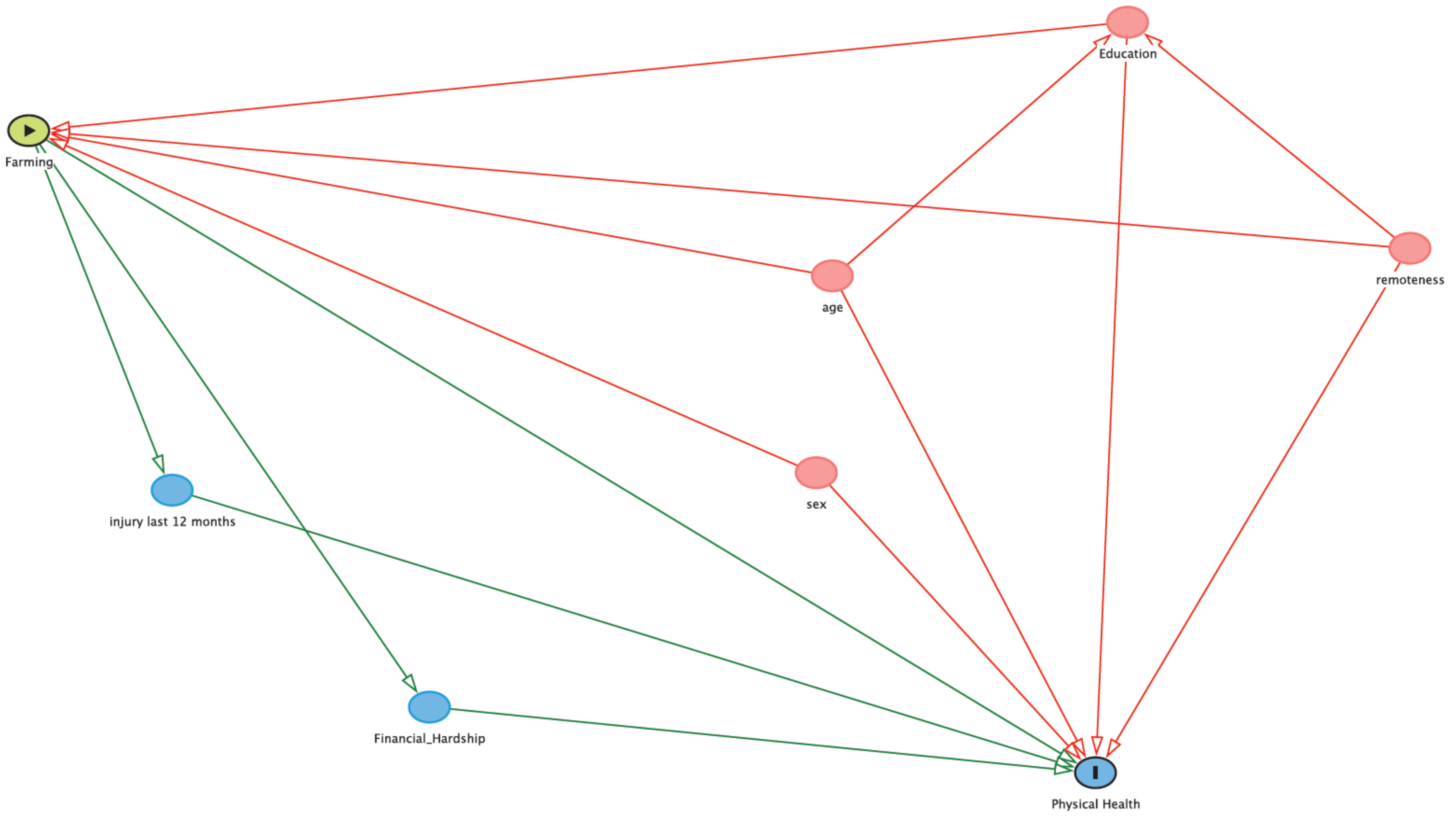

Figure 3. Directed Acyclic Graph for Farming and Wellbeing

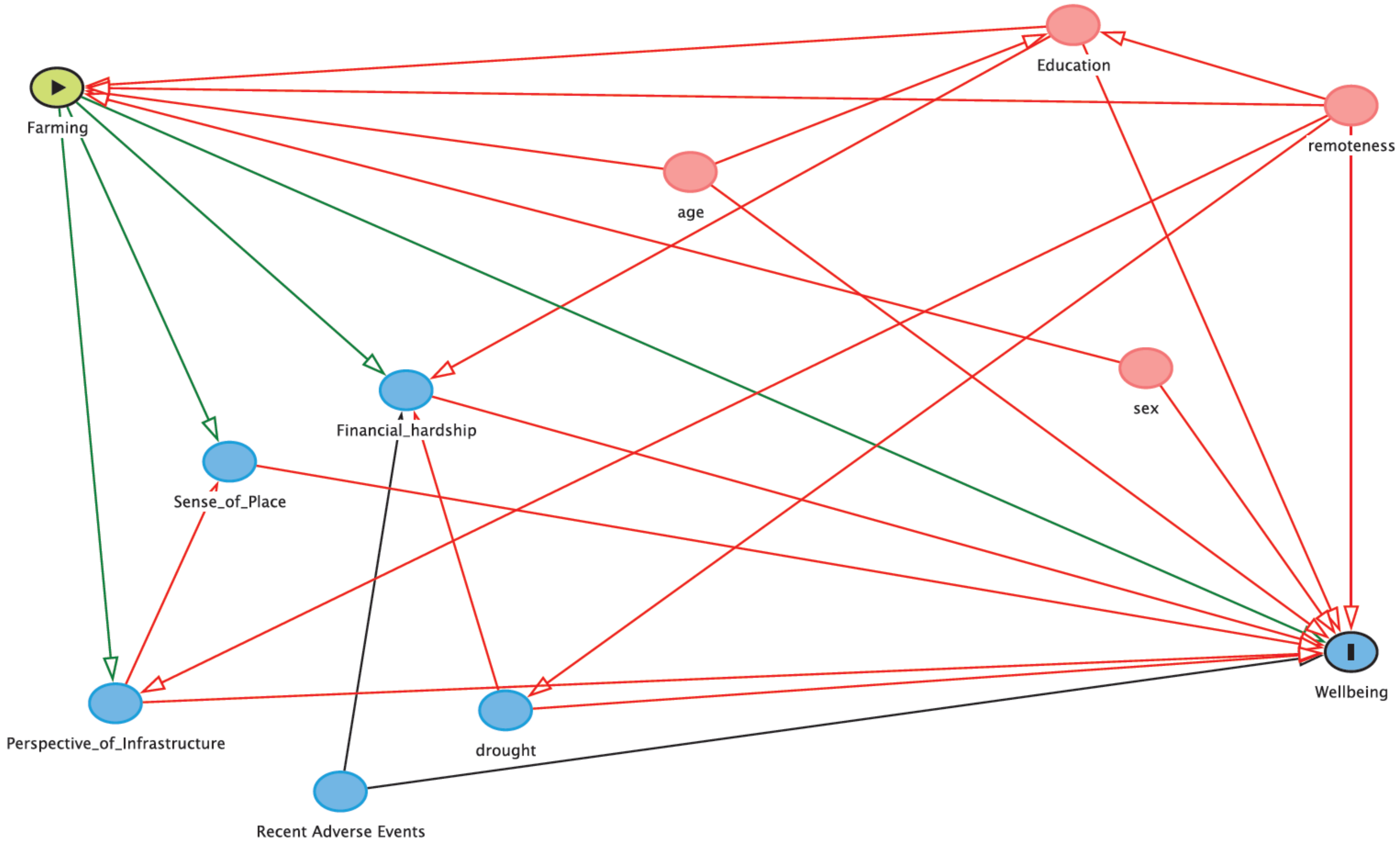

Figure 4. Directed Acyclic Graphs for Farming and Visits to the Doctor.

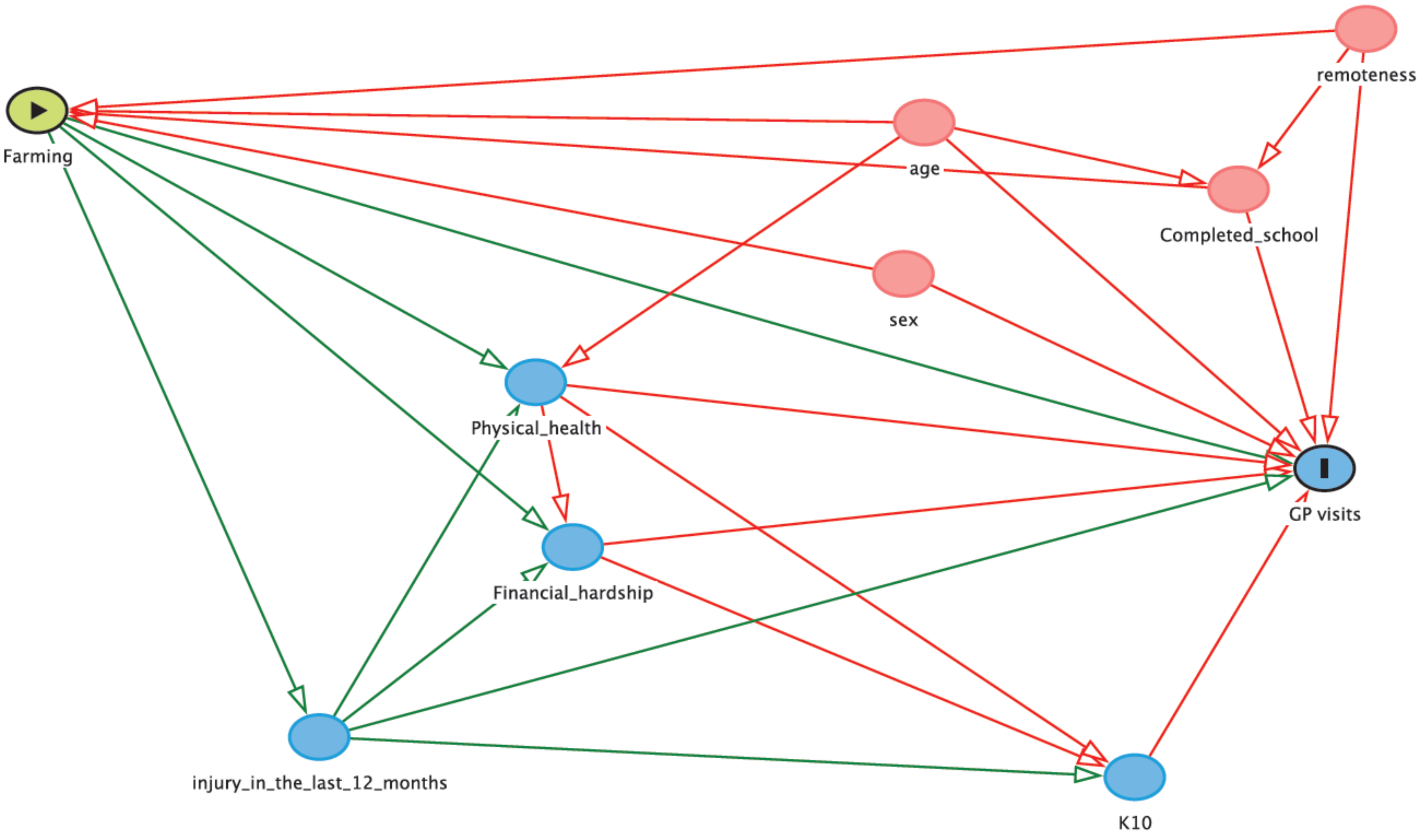

Figure 5. Directed Acyclic Graph for Farming and Seeking Help from a Mental Health Professional

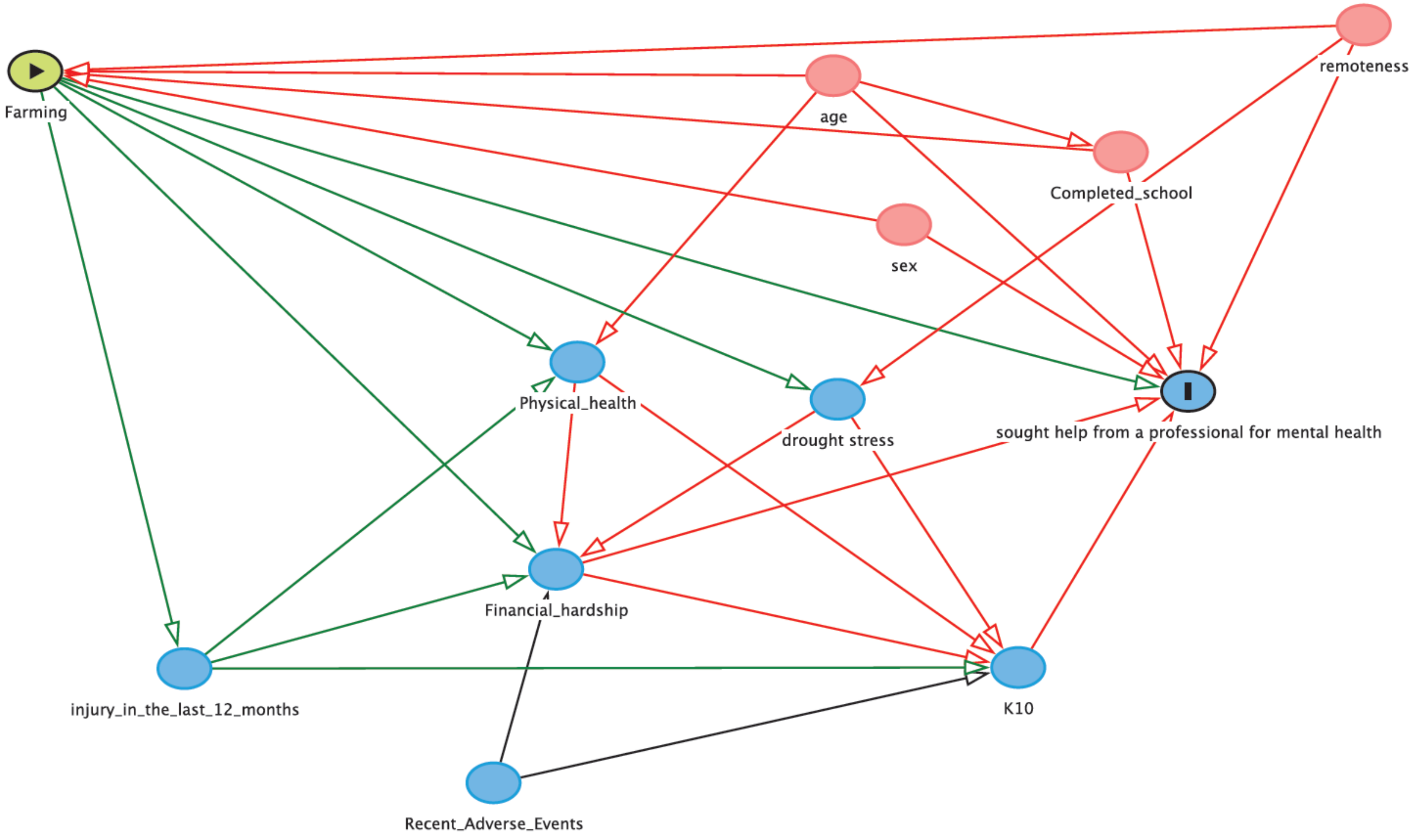

Table 1. DIRECT effect. Longitudinal analysis over 5 years for Physical health outcomes and GP service use. Farmers v's Non-Farm workers. Results show Beta values for continous variables and odds ratios for dichotomous variables (95% Confidence Intervals).

	**DIRECT effect[a]<br>GEE Farmer v Non-Farm Worker**	
	**ASGC Regional (Inner and Outer)**	**ASGC Remote (remote and very remote)**
**Self-report Physical Health N=2599**	-0.04 (-0.22, 0.14) p=0.69	-0.17 (-0.38, 0.04) p=0.10
**Alcohol Consumption AUDIT N=2595**	β = -0.40 (-1.1, 0.30)	
**Current Smoker N=2597**	OR 0.61 (0.33, 1.12)	
**Overweight BMI≥25 N=1808**	OR 0.79 (0.52, 1.22)	
**Chronic Illness N=2608**	OR 0.66 (0.43, 1.02) p=0.06	
**Injury in the last 12 months N=2615**	OR 1.04 (0.73, 1.49)	
**Number of visits to a GP in the last 12 months N=2534**	β = **-0.16 (-0.27, -0.04) p< 0.01**	

[a] DIRECT effect adjusted for confounders (age, gender, ARIA, completed school) and **mediators** according to DAGs: Physical Health outcomes- injury in the last 12 months and financial hardship (only financial hardship applied for Injury in last 12 months and Chronic illness outcomes). GP visits- financial hardship, physical health, injury in the last 12 months

Table 2. DIRECT effect. Longitudinal analysis over 5 years for Wellbeing, Mental health outcomes and Visiting a Mental Health Professional. Farmers v's Non-Farm workers. Results show Beta values for continuous variables and odds ratios for dichotomous variables (95% Confidence Intervals).

	**DIRECT effect[a] GEE Farmer v Non-Farm Worker**	
	**ASGC regional (inner or outer)**	**ASGC remote (remote or very remote)**
**Wellbeing Index N=1417**	β = -0.10 (-0.24, 0.04)	**β = -0.22 (-0.38, -0.06) p<0.01**
**Self-report Mental Health N=1404**	β = -0.03 (-0.21, 0.16)	**β = -0.19 (-0.37, -0.01) p=0.04**
**Psychological distress K10 N=1400**	β = 0.01 (-0.04, 0.05)	
**Current depression PHQ-9 N=1395**	β= -0.18 (-0.66, 0.30)	
**Sought help from a Mental Health professional in last 12 months N=2639**	**OR 0.54 (0.31, 0.93) p=0.03**	

[a] DIRECT effect adjusted for confounders (age, gender, ARIA, completed school) and **mediators** according to DAGs: Wellbeing- financial hardship, infrastructure perception, recent adverse events, sense of place, worry about drought; Mental health outcomes- community and personal support, financial hardship, infrastructure perception, self-report physical health, recent adverse events, worry about drought, injury in last 12 months; Mental health professional visits- K10, financial hardship.
